# High‐Dose Continuous Infusion Ifosfamide as Effective Palliation in a Patient With Relapsed Ewing Sarcoma With Bone Marrow Infiltration and Severe Thrombocytopenia: A Case Report

**DOI:** 10.1002/cnr2.70468

**Published:** 2026-02-18

**Authors:** Fabio Murtas, Benedetta Chiusole, Ilaria Tortorelli, Silvia Finotto, Marta Burei, Marina Coppola, Antonella Galiano, Maital Bolshinsky, Elena Bellan, Marta Sbaraglia, Angelo Paolo Dei Tos, Antonella Brunello

**Affiliations:** ^1^ Oncology 1 Unit, Department of Oncology Istituto Oncologico Veneto IOV – IRCCS Padua Italy; ^2^ Department of Surgery, Oncology and Gastroenterology (DISCOG) University of Padua Padua Italy; ^3^ Department of Nuclear Medicine, Veneto Institute of Oncology IOV‐IRCCS Padua Italy; ^4^ Pharmacy Veneto Institute of Oncology IOV‐ IRCCS Padua Italy; ^5^ Surgical Pathology and Cytopathology Unit University Hospital of Padua Padua Italy; ^6^ Department of Medicine University of Padua School of Medicine Padua Italy

**Keywords:** bone marrow, chemotherapy, ifosfamide, sarcoma, thrombocytopenia

## Abstract

**Background:**

Ewing sarcoma is a rare primary mesenchymal tumor of the bone that requires an intensive multimodal therapeutic approach. Multidrug chemotherapy regimens are also the backbone for relapsing/recurring Ewing sarcoma treatment, yet when the disease relapses as bone marrow infiltration, combination chemotherapy might be difficult to administer and prognosis is poor.

**Case:**

This report describes the case of a 22‐year‐old patient with Ewing sarcoma who developed severe pancytopenia due to bone marrow infiltration, and who was treated with high‐dose continuous infusion ifosfamide, obtaining both clinical, radiological, and hematological response lasting for about 7 months.

**Conclusion:**

To our knowledge, this is the first described case of a patient with bone marrow infiltration from Ewing sarcoma presenting with severe thrombocytopenia successfully managed with low‐dose continuous infusion ifosfamide, providing almost 7 months of progression‐free survival. Considering the very dismal prognosis of Ewing sarcoma relapsing with bone marrow infiltration, this case might be of help when decision‐making is required in this setting.

## 1 Introduction

Ewing sarcoma is the third most common bone sarcoma and the second most common malignant bone tumor in children and young adults, with an incidence of 0.1/100 000/year and a median age at diagnosis of 15 years. Most cases arise in the extremities, but axial skeleton and soft tissue origin are possible [1]. Histological diagnosis is supported by the detection of specific gene translocations, generally resulting in EWSR1‐FLI1 fusion (EWS‐RNA binding protein 1—Friend leukemia integration 1 transcription factor) or, more rarely, in other ETS genes (Erythroblast Transformation‐Specific) such as EWSR1‐ERG fusion (ETS Related Gene) [2]. With the introduction of multiagent perioperative chemotherapy, including vincristine, doxorubicin, cyclophosphamide, ifosfamide and etoposide, 5‐year OS increased to 65%–70% for localized disease [1]. About 25% of patients present with advanced disease at first diagnosis: most frequently involved sites are lung, bone and bone marrow. For this reason, an accurate skeletal staging with positron emission tomography (PET‐CT) is mandatory and, in doubtful cases, this should be completed with bone marrow biopsy and aspirate [3, 4]. Early relapsed disease is characterized by a poor prognosis [5] and, if bone marrow involvement is present, chemotherapy is mainly administered with a palliative intent.

Indeed, Ewing sarcoma recurrence with bone marrow involvement has been associated with very poor survival and unavoidable fatal outcome, in contrast to patients with multiple bone metastases but no marrow involvement [6]. High‐dose ifosfamide has been recently demonstrated to be an effective regimen for recurrent disease, with other active regimens being cyclophosphamide and topotecan, temozolomide and irinotecan, and gemcitabine and docetaxel [7]. Here we present the case of a young male patient affected by extremity Ewing sarcoma recurring 15 months after the end of adjuvant chemotherapy with bone marrow infiltration causing severe pancytopenia.

## 2 Case Report

**FIGURE 1:**
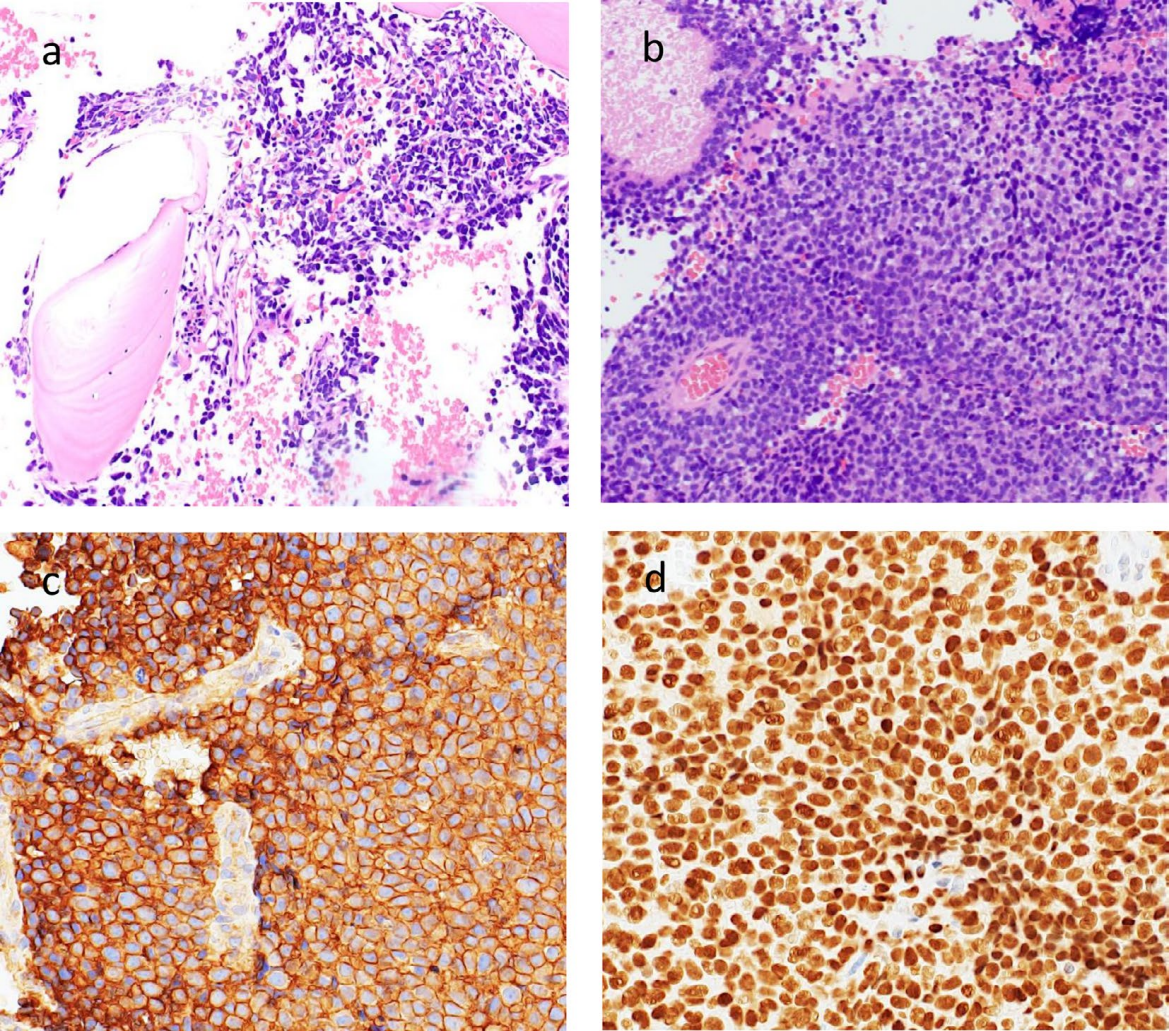
H&E bone marrow biopsy showing extensive infiltration by Ewing sarcoma. Small round cells with scant cytoplasm arranged in sheets fill the marrow space (A and B). Strong membranous CD99 (C) and diffused NKX2.2 (D) immunopositivity (magnification ×20).

**FIGURE 2:**
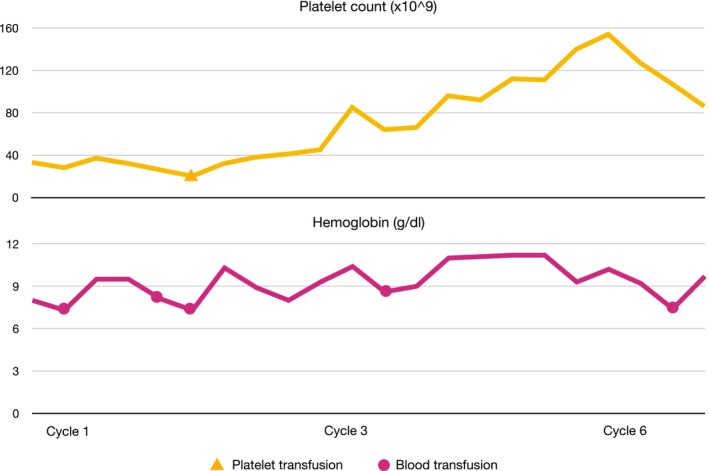
Platelet and hemoglobin levels during treatment with HD‐ifosfamide and required transfusions. Blood tests progressively improved during treatment until progression after cycle 6 when platelet count abruptly dropped.

**FIGURE 3:**
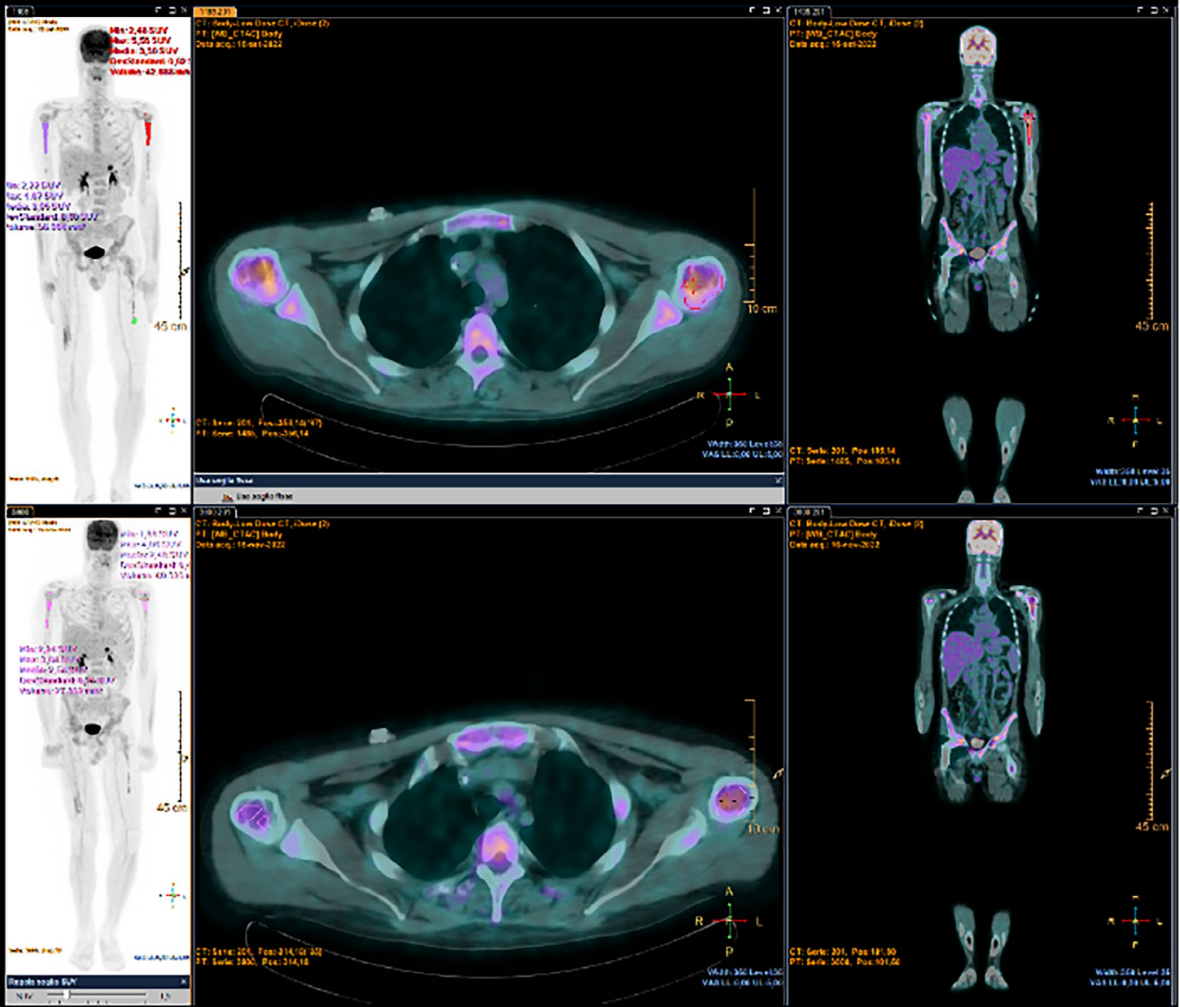
PET‐CT performed before (a) and after (b) three cycles of HD‐ifosfamide, showing partial metabolic response to treatment on bone marrow infiltration.

In April 2019, a 22‐year‐old male with no past medical history presented with increasing pain in the right pelvis and inferior limb. Pain was at first managed by his general practitioner with nonsteroidal anti‐inflammatory drugs (NSAIDs) and steroids with no clear clinical benefit. In February 2020, a magnetic resonance (MRI) of the right hip was performed, which detected a 9 cm lesion extending from the femoral head to the bone neck, hyperintense on T2‐weighted images suggesting intraspongious edema. A computed‐tomography (CT) scan subsequently performed confirmed the presence of a vast, irregular osteolytic area and a hyperdense tissue in the great trochanter region, with a maximum depth of 15 mm. In March 2020, a biopsy of the osteolytic area was performed, and the histological specimen showed a proliferation of undifferentiated, small round cell positive for CD99, FLI1, vimentin and focal CD56, suggestive of Ewing sarcoma. The molecular analysis confirmed the presence of EWSR1‐ERG rearrangement (21q22.2). Staging chest and abdomen CT and PET‐CT scan did not detect any distant metastases; right femur head, neck, intertrochanteric region and proximal diaphysis showed a pathological hypermetabolism (SUV max 7.16), but bone marrow fine needle aspiration was negative for neoplastic cells. From April to June 2020, patient received four courses of induction chemotherapy: two cycles of vincristine, doxorubicin and cyclophosphamide (VDC), one cycle of vincristine, actinomycin‐D and ifosfamide (VAI) and one cycle of etoposide and ifosfamide (IE). The following radiological assessment (chest, abdomen and right inferior limb CT scan and right hip MRI) showed disease stability. In July 2020, patient underwent surgical resection of right femur and global modular replacement system prosthesis implant. Pathological response was good, although not complete, with a 95% necrosis rate and 5% of residual vital cells. After surgery, the patient experienced slowly increasing pain at his right thigh, which was refractory to analgesics and opioids. Surgery was followed by adjuvant chemotherapy: 3 cycles of VDC, 3 cycles of VAI and 3 cycles of IE. Pain on his right thigh subsequently started to improve. At the end of adjuvant chemotherapy follow‐up was started with trimestral chest, abdomen and right inferior limb CT scan and blood tests. Radiological assessments did not detect any signs of disease relapse; blood samples showed persistently stable G2 neutropenia (N 1380/mm^3^), G1 anemia (Hb 13.9 g/dL) and G1 lymphocytopenia (LYMPH 0.98/mm^3^), consistent with recent chemotherapy. After one year, in August 2022, blood tests showed pancytopenia, with G1 neutropenia (N 1750/mm^3^), G1 lymphocytopenia (LYMPH 0.97/mm^3^), G2 thrombocytopenia (PLT 74000/mm^3^) and G1 anemia (11.4 g/dL). Lactate dehydrogenase (655 U/L) and ferritin (1505 ug/l) levels were increased. Peripheral blood smear identified the presence of immature cells (myelocytes 4%, metamyelocytes 0.5%). Lymphocytes flow cytometry analysis, vitamin B12, protein electrophoresis, alkaline phosphatase, renal and hepatic function were preserved. The patient reported the onset of widespread bone pain, especially at ribs, cranium and sternum. At this time, MRI of right inferior limb displayed intraspongious edema of ilio‐pubic branch, CT scan was persistently negative. No signs of recent bleeding were observed. A bone marrow fine needle aspiration and a biopsy were then performed. The myelogram showed the presence of big, pleomorphic non hematopoietic cells. Ewing sarcoma bone marrow relapse was confirmed by the biopsy, where most bone marrow space was occupied by a high‐grade neoplasm composed of small round cells, arranged in sheets, characterized by monotonous nuclear appearance and scant cytoplasm with focal areas of cytoplasmic clearing. Brisk mitotic activity, areas of hemorrhage and necrosis were present throughout the biopsy specimen. The neoplastic cells showed strong crisp membranous CD99 immunostaining and diffuse NKX2.2 nuclear immunopositivity. Conversely, terminal deoxynucleotidyl transferase (TdT), desmin and myogenin (Myf4) were negative (Figure 1). A PET‐CT was performed which showed an intense bone marrow glucidic hypermetabolism in spine, scapulae, sternum, ribs, hipbone, humeri and femurs. At the end of August 2022 blood cell counts rapidly dropped, with severe anemia (Hb 8 g/dL) and thrombocytopenia (PLT 33000/mm^3^). Given the low blood cell counts which contraindicated standard poly‐chemotherapy regimens, we decided to start first line chemotherapy with high dose ifosfamide at the dose of 1 g/m^2^ per day, administered as a continuous infusion over 14 days, followed by a 14 days pause. The first course of such chemotherapy was started in August 2022, with a 50% precautionary dose‐reduction (total dose: 7 g/m^2^ over 2 weeks). A strict, daily monitoring of blood count was performed both during infusion and in the 2 weeks off; toxicity nadir was reached on day 17, with G3 anemia (Hb 7.3 g/dL) and G4 thrombocytopenia (PLT 21000/mm^3^). Persistent hematological toxicity required one platelet and multiple red blood cells transfusions. At the beginning of the second course, blood counts improved with neutrophil count back to normal, improved hemoglobin (Hb 10.3 g/dL) and lymphocyte count (LYMPH 0.82/mm^3^) but persisting severe thrombocytopenia (32 000/mm^3^). Following the second course, administered at the same reduced dose, platelet count started to slowly increase; no platelet and less red blood cell transfusions were needed. On the third cycle, thrombocytopenia was restored to G1 level (85 000/mm^3^), and chemotherapy dosage was increased to 80% (total dose 11 g/m^2^), with a good hematological and subjective tolerance (Figure 2). After three cycles, PET‐CT scan showed metabolic response (Figure 3). Due to the persistently improved blood tests, and the normalization of platelet count, chemotherapy was continued at full dose for three more cycles. Metabolic and hematological responses were associated with clinical benefit on pain and deambulation. After six total courses of high‐dose ifosfamide, PET‐CT showed progressive bone marrow disease and once again blood tests dropped, with G3 neutropenia, G2 lymphocytopenia, G1 anemia and G1 thrombocytopenia. Moreover, the patient developed vision problems, hyposthenia and hypoesthesia of lower limbs, and a brain MRI demonstrated a diffuse meningeal invasion. Therefore, a new line of chemotherapy with Irinotecan and Temozolomide was started. Although it was administered at a reduced dose, therapy was complicated by persistent G3 neutropenia needing frequent treatment delays and dose adjustments. After six cycles, the patient was hospitalized due to paraparesis, acute urinary retention and pain; a CT scan confirmed new bone progression. He received palliative radiotherapy on painful sites and was started on oral etoposide with only small benefit. Palliative care team was then involved, and the patient died 2 months later at home.

## 3 Discussion

Thrombocytopenia is a frequent event in oncological patients. Its incidence is estimated to range from 10% to 68%, according to different studies [8]. About 21% of patients with hematologic malignancies and up to 6% of patients with solid tumors present thrombocytopenia before chemotherapy. At diagnosis, the most frequently associated histotypes are acute leukemia (37.3%) and multiple myeloma (24.4%); among solid tumors, melanoma (21.4%), ovarian (14.7%), and lung (14.3%) cancer [9]. In a recent retrospective study, cancers of unknown origin, lung, soft tissue tumors, stomach, nasopharynx, and breast cancer were most commonly associated with bone marrow infiltration [10]. After 3 months of chemotherapy, the reported incidence rates increase to 28.2% and 12.8% for hematological and solid neoplasms, respectively [9]. High‐dose cyclophosphamide and doxorubicin, commonly used in the treatment of sarcomas, have been associated with an increased need for platelet transfusions during therapy [11].

While most cases are asymptomatic, a decrease in platelet count may lead to minor and major bleeding, especially when platelet counts drop to grade 2 and lower. A study by Elting and colleagues demonstrated how bleeding episodes complicated 9% of chemotherapy cycles in patients with a history of previous bleeding and who had baseline platelet count lower than 75 000/mm^3^, bone marrow metastases or poor performance status [12].

No clear guidelines on management of patients with bone marrow infiltration are available: most data are obtained from case reports or small retrospective studies of patients treated with different cytotoxic agents according to histology, with great heterogeneity on outcomes. A French retrospective study showed the efficacy and feasibility of weekly, low dose paclitaxel in 26 patients with breast cancer bone marrow infiltration [13]. Similarly, analogous cases of breast cancer bone marrow infiltration have been successfully treated with continuous infusion doxorubicin [14] or weekly nab‐paclitaxel [15]. On the other hand, a patient with neuroblastoma did not derive any benefit from combination therapy of standard dose topotecan and cyclophosphamide [16].

Among mesenchymal tumors, bone marrow infiltration in Ewing sarcoma and pediatric rhabdomyosarcoma is not uncommon [17, 18]; more rarely, bone marrow infiltration from other histologic subtypes like angiosarcoma [19], epithelioid sarcoma [20] and follicular dendritic cell sarcoma [21] has been reported. Given the aggressiveness of such sarcoma subtypes, most cases were treated with high dose, multidrug regimens with poor responses and severe hematological toxicity, often fatal [22].

Despite the absence of data on efficacy of chemotherapy in bone marrow infiltration of Ewing sarcoma, its palliative role should be discussed case by case. Chemotherapy, acting on the very first cause of thrombocytopenia, surely has a leading role in the treatment of bone marrow infiltration. All the reported successful cases have in common the administration of low dose, continuous infusion or weekly fractionated chemotherapy regimens, with the aim of reducing the negative impact on hematopoietic bone marrow cells. We decided to use the above‐mentioned regimen taking into account the available data on low dose chemotherapy in several cancer histotypes, and the data on reduced toxicity of continuous infusion versus bolus ifosfamide. In fact, data from Plutt et al. [23] show that patients with Ewing sarcoma treated with continuous infusion ifosfamide had lower incidence of hematological toxicities compared to those who received bolus infusion. Other effective chemotherapy regimens like temozolomide and irinotecan, which are commonly used in relapsed Ewing sarcoma [24], might be more challenging to resort to due to their known toxicity profile: temozolomide has a typical hematological toxicity, with not negligible rates of neutropenia and thrombocytopenia, that are not ideal in the case of bone marrow infiltration. Moreover, our decision was corroborated by the recently published results of rEEcur study, which demonstrated the effectiveness of a high dose ifosfamide in refractory/relapsed Ewing sarcoma [7].

Given the lack of historical control, we cannot demonstrate a benefit on survival for our patient; nevertheless, its role in palliation and symptomatic relief was clear.

## 4 Conclusion

To our knowledge, this is the first described case of a patient with bone marrow infiltration from Ewing sarcoma presenting with severe thrombocytopenia successfully managed with low‐dose continuous infusion ifosfamide, providing almost 7 months of progression‐free survival.

Considering the very dismal prognosis of Ewing sarcoma relapsing with bone marrow infiltration, this case might be of help when decision‐making is required in this setting.

## Author Contributions

Fabio Murtas wrote the original draft. Antonella Brunello and Benedetta Chiusole reviewed and edited the original draft. Fabio Murtas, Benedetta Chiusole, Ilaria Tortorelli, Silvia Finotto, Marta Burei, Marina Coppola, Antonella Galiano, Maital Bolshinsky, Elena Bellan, Marta Sbaraglia, Angelo Paolo Dei Tos, and Antonella Brunello were involved in data curation and treatment of the patient. All authors read and approved the final written manuscript.

## Funding

The authors have nothing to report.

## Ethics Statement

The study was conducted in accordance with institutional ethical standards.

## Consent

Patient's relatives gave consent for publication.

## Conflicts of Interest

The authors declare no conflicts of interest.

## Data Availability

Data sharing not applicable to this article as no datasets were generated or analysed during the current study.
